# Stair-Step Pattern of Soil Bacterial Diversity Mainly Driven by pH and Vegetation Types Along the Elevational Gradients of Gongga Mountain, China

**DOI:** 10.3389/fmicb.2018.00569

**Published:** 2018-03-27

**Authors:** Jiabao Li, Zehao Shen, Chaonan Li, Yongping Kou, Yansu Wang, Bo Tu, Shiheng Zhang, Xiangzhen Li

**Affiliations:** ^1^Key Laboratory of Environmental and Applied Microbiology, Chengdu Institute of Biology, Chinese Academy of Sciences, Chengdu, China; ^2^Environmental Microbiology Key Laboratory of Sichuan Province, Chengdu, China; ^3^Department of Ecology, Key Laboratory of Ministry of Education for Earth Surface Processes, Peking University, Beijing, China

**Keywords:** stair-step pattern, bacterial diversity, community assembly, environmental filtering, spatial attributes, elevational gradients, Gongga Mountain

## Abstract

Ecological understandings of soil bacterial community succession and assembly mechanism along elevational gradients in mountains remain not well understood. Here, by employing the high-throughput sequencing technique, we systematically examined soil bacterial diversity patterns, the driving factors, and community assembly mechanisms along the elevational gradients of 1800–4100 m on Gongga Mountain in China. Soil bacterial diversity showed an extraordinary stair-step pattern along the elevational gradients. There was an abrupt decrease of bacterial diversity between 2600 and 2800 m, while no significant change at either lower (1800–2600 m) or higher (2800–4100 m) elevations, which coincided with the variation in soil pH. In addition, the community structure differed significantly between the lower and higher elevations, which could be primarily attributed to shifts in soil pH and vegetation types. Although there was no direct effect of MAP and MAT on bacterial community structure, our partial least squares path modeling analysis indicated that bacterial communities were indirectly influenced by climate via the effect on vegetation and the derived effect on soil properties. As for bacterial community assembly mechanisms, the null model analysis suggested that environmental filtering played an overwhelming role in the assembly of bacterial communities in this region. In addition, variation partition analysis indicated that, at lower elevations, environmental attributes explained much larger fraction of the β-deviation than spatial attributes, while spatial attributes increased their contributions at higher elevations. Our results highlight the importance of environmental filtering, as well as elevation-related spatial attributes in structuring soil bacterial communities in mountain ecosystems.

## Introduction

Unraveling the drivers and mechanisms of community succession and community assembly along ecological gradients are two major goals in ecology (Chase, 2003; Zhou et al., 2014). The mountain ecosystems represent one of the most important components in the terrestrial system, and provide significant ecological services. In mountain ecosystems, climate, vegetation and soil properties vary drastically over short spatial distance (McCain and Grytnes, 2010). Therefore, elevational gradients can serve as a natural platform to reveal potential microbial responses to climate change with a strategy of space-for-time substitution (Dunne et al., 2004). To date, elevational biodiversity patterns for macro-organisms have been extensively recorded (McCain and Grytnes, 2010), which shows maximum diversity at lower or middle elevations. However, the elevational diversity patterns of microbial communities appear to be more divergent, such as increasing (Wang J. et al., 2017), decreasing (Bryant et al., 2008; Shen et al., 2015; Wang J.-T. et al., 2015), and mid-elevation patterns (Singh et al., 2012a,b), or no consistent pattern (Fierer et al., 2011; Shen et al., 2013). Thus, the elevational patterns of soil microbial communities in a mountain ecosystem remain controversial.

Many environmental factors that co-vary with elevation may regulate specific microbial assemblages and therefore cooperate in shaping the overall microbial community compositions (Sundqvist et al., 2013). A variety of studies indicate that soil bacterial communities are primarily shaped by soil pH across a variety of ecosystems (Fierer and Jackson, 2006; Rousk et al., 2010; Kraft et al., 2015; Tripathi et al., 2015; Xia et al., 2016). However, Shen et al. (2013, 2015) report that factors shaping soil bacterial communities differ in elevational scales even in a single mountain, with carbon and nitrogen as the key drivers at a smaller scale (2000–2500 m), while soil pH as the key driver at larger elevational gradients (530–2200 m). Besides, vegetation types could play important roles in shaping soil bacterial communities (Chu et al., 2011; Zhang et al., 2015). Different plant communities could result in differential microbial assemblages in the soil (Prober et al., 2015), either through the rhizodeposits or by changing soil conditions (Wardle et al., 2004; De Deyn and Van der Putten, 2005). In addition, other studies also revealed temperature as the major factor in controlling microbial elevational diversity patterns (Nottingham et al., unpublished). Therefore, interactions of multiple factors along the elevational gradients may further complicate the relative roles of different environmental drivers in soil bacterial community succession.

Understanding mechanisms of microbial community assembly is a central issue in community ecology (Chase, 2003; Zhou et al., 2014). Although many studies have been carried out to elucidate microbial assembly mechanisms across a wide range of ecosystems (Myers et al., 2013; Wang et al., 2016), the mechanisms of elevational community assembly of soil microbes remain elusive. Niche and neutral processes represent two models accounting for microbial assembly (Hubbell, 2001; Mikkelson, 2005). Currently, increasing evidences suggest that both niche and neutral processes concurrently guide microbial community assembly with varying relative contributions largely depending on geographic scales (Hanson et al., 2012; Bahram et al., 2016). For example, spatial distances are reported to better account for microbial assemblages at large scales (Shi et al., 2015; Wang X. et al., 2015), while environmental attributes play more important roles at small scales (Horner-Devine et al., 2004; Hollister et al., 2010). Moreover, it is reported that the relative importance of these processes varies across habitat types (Wang X.-B. et al., 2017). Geographic distance is solely responsible for soil community similarity in desert, whereas microbes community similarity is controlled by environmental factors in the alpine grassland (Wang X.-B. et al., 2017). Because elevation is the most dominant spatial character in mountain ecosystems, different elevation level may lead to differential contributions of environmental and spatial attributes to microbial communities. Further, vegetation vary drastically along the elevational gradients (McCain and Grytnes, 2010), which may modulate their relative contributions as well. Therefore, to understand the assembly mechanisms of soil microbiota in mountain ecosystems, it is necessary to partition the variation in species composition into fractions explained by the effect of spatial versus environmental attributes.

The Gongga Mountain is the highest mountain in the east boundary of the Tibet Plateau (Wu et al., 2013), which has attracted numerous studies on elevation patterns of the climate, vegetation, and biodiversity (Liu, 1985; Shen et al., 2001, 2004). In this study, we looked into the composition and diversity of soil bacteria along the elevational gradients from 1800 to 4100 m in Gongga Mountain. The objectives of this study were mainly to reveal (1) soil bacterial diversity patterns and their environmental drivers and (2) the mechanism of bacterial community assembly along the elevational gradients.

## Materials and Methods

### Site Description

The Gongga Mountain is located in the west part of Sichuan Province, Southwest China (ca. 29°01’–30°05’ N, 101°29’–102°12’ E). With the peak height of 7556 m, Gongga Mountain is the summit of the Hengduan Mountain Ranges, which constitutes the eastern boundary of the Tibetan plateau. Located at the core part of the Hengduan Mountain Ranges, one of a global biodiversity hotspot, Gongga Mountain extends mainly in north–south direction. Its eastern slope is deeply cut down to about 1000 m by the Dadu River, a major branch of the Yangtze River. Its western slope extends to the surface of the Tibet Plateau, cross a much shallower valley. The environment in Gongga Mountain is characterized by an extraordinarily variable topography, climate, and vegetation, virtually along the prominent elevational gradients. In July, for example, the mean monthly temperature decreases from 12.7°C at 1600 m to 4.2°C at 3000 m, whereas precipitation increases from 1050 to 1943 mm, respectively (Wu et al., 2013). Accordingly, the vegetation on the east aspect of Gongga Mountain represents the most complete vegetation spectrum along the elevational gradients of the subtropical region in China. Specifically, shrubs and grass dominate the dry valley bottom below 1200 m. In 1200–1800 m, evergreen broadleaved forests are chiefly composed of many species of *Lindera* spp., *Cinnamomum* spp., and *Cyclobanalopsis* spp. In 1800–2500 m, mixed evergreen and deciduous broadleaved forests are co-dominated by *Lithocarpus cleistocarpus* and *Quercus* spp. In 2500–2850 m, mixed forests of deciduous broadleaved and coniferous species are dominated by *Tsuga dumosa*, *Picea brachytyla*, and *Acer flabellatum*. The *Abies fabri* dominants in the subalpine forests from 2850 m up to the treeline at about 3850 m. Alpine shrubs extend from 3600 to 3700 m upward, with *Rhododendron lapponicum* dominating at the lower part. The mixed mosaic of alpine shrub and meadow spreads from 3650 to 4200 m, and alpine meadow further extends to snowline at 4900 m (Shen et al., 2004).

### Plant Survey and Soil Sampling

Plant survey was conducted along the elevational gradients of eastern slope of Gongga Mountain from May to July in 1999. Altogether 68 plots were systematically selected along the elevational gradients, from 1200 to 4500 m, with an elevation difference of 50 m between each pair of neighboring plots. The plant species composition and richness (PSR) in each plot of 20 m × 20 m area were recorded. The diameter at breast height (DBH) of each woody plant was also measured, and the total DBH for all woody plants in each elevation was summed. Because there were three types of woody plants in Gonnga Mountain, namely, deciduous broad (DB) trees, evergreen broad (EB) trees, and dark coniferous (DC) trees (Shen et al., 2001), the percentage of total DBH for each type can be calculated in each elevation. The above plant attributes are summarized in Supplementary Table S1.

We collected 99 top soil samples (0–10 cm after removing litter layer) from 12 sites along the east slope of Gongga Mountain in October 2014. These sites expanded across 1800–4100 m with a pairwise interval of approximate 200 m. In an attempt to eliminate the effect of high spatial heterogeneity on microbial communities within each sampling site, we collected four-to-five cores from 8 to 14 independent plots in each elevation (10 m × 10 m for each plot, >50 m distance between plots). Visible roots and litter were removed prior to homogenizing the soil samples. Each fresh soil sample which was passed through a 2-mm sieve was divided into two subsamples. One was stored at 4°C for soil variable measurements and the other was stored at -20°C for DNA extraction.

Soil temperature was measured in the field at the depth of 10 cm (T10) using a digital thermometer. Soil pH and electric conductivity (Cond.) were measured in the soil–water slurry (soil:water, 1:5) using a pH meter and an electric conductivity meter. The ${\mathrm{NO}}_{3}^{-}$-N and ${\mathrm{NH}}_{4}^{+}$-N were determined by the KCl extraction-colorimetric method and the indophenol blue method, respectively (Kou et al., 2017). Soil total carbon (TC) and total nitrogen (TN) were determined using an elemental analyzer (Vario Macro Cube, Germany). Precipitation for this region was linearly correlated with elevation with gradients of 74 (2000–3600 m) and 66 mm hm^-1^ (3600–6000 m), according to Cheng (1996). Therefore, mean annual precipitation (MAP) and mean annual air temperature (MAT) were linearly calculated based on the recordings at the two meteorological stations (Hailuogou Station, 101°59’54″E, 29°34’34″N, 2947.8 m and Moxi Station, 102°06’55″E, 29°38’59″N, 1621.7 m). The soil properties were summarized in Supplementary Table S2.

### Miseq Sequencing of 16S rRNA Gene Amplicons

Soil genomic DNA was extracted using PowerSoil^®^ DNA Isolation Kit (MOBIO, United States). DNA concentration and quality were determined using a ND-1000 spectrophotometer (NanoDrop, DE, United States). An aliquot (10 ng) of purified DNA from each sample was used as template for amplification. Bacterial 16S rRNA genes were amplified using universal primers 515F (5′-GTGYCAGCMGCCGCGGTA-3′) and 909R (5′-CCCCGYCAATTCMTTTRAGT-3′) (Li et al., 2014). The 12-nt unique barcode sequence was added at the 5’-end of forward primer. The following PCR conditions were used: denaturation at 94°C for 3 min, followed by 30 cycles of denaturation at 94°C for 30 s, annealing at 55°C for 1 min and extension at 72°C for 1 min, with a final extension at 72°C for 5 min. Negative controls were performed to ensure that no contamination occurred. Triplicate PCR reactions were performed per sample and pooled for purification using Gel Extraction kit (Omega Bio-Tek). Equal molar of PCR product from each sample was pooled together. The sequencing library was prepared using Truseq DNA PCR-Free Library Preparation Kits and sequenced at Illumina Miseq platform with 2 × 250 bp V2 Kits at Chengdu Institute of Biology, Chinese Academy of Sciences (CAS).

Paired-end sequences were merged using the FLASH tool (Magoč and Salzberg, 2011). The assembled sequences were demultiplexed based on the unique sample barcodes, trimmed for sequence quality, and denoized using UPARSE pipeline (Edgar, 2013). A table of operational taxonomic unit (OTU) was generated using UPARSE clustering method (97% as cutoff). OTUs with single sequences (singletons) across all samples were removed. To correct the effect of varied sequencing depth on diversity evaluation, all samples were rarefied to the same sequencing depth. Taxonomy was assigned to each OTU using the RDP Classifier with a confidence threshold of 80% (Wang et al., 2007).

### Statistical Analysis

To reveal the bacterial diversity patterns, the number of OTUs (richness) and phylogenetic diversity (Faith’s PD) were compared among the sampling sites using Kruskal–Wallis and associated multiple comparison tests. To determine bacterial abundance in each sample, quantitative PCR (QPCR) was carried out with the same primers used in Miseq sequencing of 16S rRNA gene amplicons. Nonmetric multidimensional scaling (NMDS) analysis based on Jaccard or Bray-Curtis distances was conducted to perceive the changes of bacterial community structure along the elevational gradients. The two matrices resulted in similar results (Supplementary Figure S1). Nonparametric tests including Adonis analysis of similarities (ANOSIM) and multiple-response permutation procedure (MRPP) tests were performed to compare community dissimilarities. Spearman rank correlation analyses were carried out to depict the relationships between environmental factors and α-diversity and the relative abundance of bacterial lineages. Partial Mantel tests were applied to reveal relationships between bacterial community structure and environmental attributes. Canonical correspondence analysis (CCA) with forward-model selection (“forward.sel” function) was applied to infer the relative importance of environmental attributes to community compositions. All above statistics were performed using the “pgirmess,” “vegan,” “psych,” and “packfor” packages of R program (R Core Team, 2016).

### Partial Least Squares Path Modeling (PLS-PM)

Partial least squares path modeling (PLS-PM) were performed to further reveal the possible pathways through which environmental attributes guide bacterial composition succession along the spatial gradients (Sanchez, 2013). We selected the best models where all paths were statistically significant. PLS-PM analysis is an approach to study observed variables that can be summarized by the use of a latent variable (Sanchez, 2013). The observed variables in the PLS-PM were latent variables. The latent variables in the PLS-PM studied here included climate, plant, soil attributes, and bacterial communities. Each latent variable could include one or more manifest variables. Since most environmental attributes were significantly correlated to each other, all the environmental attributes were applied in the model. For example, MAT and MAP for climate; PSR and DB for plant; pH and electric conductivity for soil attributes; the first (NMDS1) and second (NMDS2) axis of NMDS for bacterial communities. Path coefficients (representing the direction and strength of linear relationships between latent variables) and explained variability (*R*^2^) were estimated by models. Models with different structures were evaluated using the goodness of fit (GOF) statistic, a measure of the overall predictive power, and GOF > 0.7 is the acceptable value for the PLS-PM (Sanchez, 2013). The model was constructed using the function “innerplot” in “plspm” R package (Sanchez, 2013).

### Null Model Analysis

β-Diversity, representing the variation between communities, provides critical inference to the mechanisms of community assembly, such as the contribution of niche versus neutral process. However, the variation of β-diversity could simply be due to the variations in α- or γ-diversity (Chase et al., 2011). Thus, a null model approach is required to discern whether the variation is more accounted for by ecological processes, or instead by the difference in α-diversity or experimental treatments. If the observed β-diversity was significantly deviated from the random null expectations, deterministic processes such as environmental filtering could play dominant roles in structuring microbial communities. Otherwise, stochastic processes could contribute more to the community assembly. Here, the regional species pool is defined as the total number of OTUs observed in all collected soil samples.

A Jaccard metric was used to assess whether the number of shared species between any two communities were different from the null expectation (Zhou et al., 2014). The observed similarity (*J*_obs_) and average expected similarity (*J*_exp_) of 999 iterations were obtained through the null model analysis. The permutation analysis of multivariate dispersions (PERMDISP) based on distance-to-centroid values was performed to test the significance of differences in microbial communities within each elevational site from null model expectations (Anderson, 2006). From 999 iterations, we calculated the β-deviation, which was defined as the difference between observed and mean expected Jaccard’s similarity divided by the standard deviation of expected values. β-Deviation represented the β-diversity after controlling sampling effects (Myers et al., 2013). The above analyses were carried out with the “vegan” package of R programs.

### Distance-Based Redundancy Analysis (dbRDA)

To account for the variation in β-deviation, we used distance-based redundancy analysis (dbRDA) (“capscale” function) to partition the variation of β-deviation into fractions explained by environmental and spatial attributes (Peres-Neto et al., 2006; Legendre et al., 2009). Our initial environmental attributes encompassed seven soil properties and two climate variables (Supplementary Table S2). Spatial attributes included the latitude, longitude, and elevation of each plot, as well as positive eigen-values obtained from principal components of neighbor matrices (PCNM) using “pcnm” function in “vegan” package (Peres-Neto et al., 2006; Legendre et al., 2009). Because both our α-diversity and NMDS plots of community structure showed two distinct sections separated by elevation (lower: 1800–2600 m; higher: 2800–4100 m), this suggests that the underlying mechanisms of community assembly differ in lower and higher elevational sections. Thus, further comparison was carried out between the two elevational sections.

Given the collinearity detected among the involved variables, we first tested the significance of a full model including all the explanatory variables, and then simplified the model using forward-model selection procedure (“ordiR2step” function) (Blanchet et al., 2008). The retained explanatory variables were used to partition the variation of β-deviation into four components: pure environmental effect, pure spatial effect, spatially structured environmental effect, and the unexplained residual. Finally, we tested the significance of fractions explained by environmental attributes and spatial attributes in both elevation sections, using bootstrap test in Octave 4.0 (999 iterations) (Peres-Neto et al., 2006).

### Nucleotide Sequence Accession Number

The original sequencing data are available at the European Nucleotide Archive by Accession No. PRJEB15866^1^.

## Results

### Climate and Soil Properties Along the Elevational Gradients

In this study, MAP increased, while MAT and T10 showed decreasing trends with elevation (Supplementary Table S2). TC, TN, ${\mathrm{NH}}_{4}^{+}$-N, and ${\mathrm{NO}}_{3}^{-}$-N varied from 0.60 to 37.54%, 0.08 to 2.24%, 1.81 to 62.78 mg (kg dry wt soil)^-1^, and 0 to 25.33 mg (kg dry wt soil)^-1^, respectively. The soil electric conductivity ranged from 0 to 262 μS cm^-1^. Soil pH value varied from 3.53 to 7.23, and pH 6.0 was the threshold that distinguished the lower (1800–2600 m) from higher (2800–4100 m) elevations. Regarding to plant attributes, plant species richness (PSR) generally decreased with elevation (Supplementary Table S1). Among the woody plant species, the percentage of total DBH for DB trees, EB trees, and DC trees also showed different elevational changes. Spearman’s correlation analysis indicated that most environmental attributes were significantly correlated to each other (Supplementary Table S3).

### Bacterial Community Compositions and α-Diversity

A total of 4,290,576 sequences were identified from all the 99 soil samples, ranging from 9589 to 128,799 reads per sample. UPARSE pipeline resulted in a total of 3220 OTUs using an arbitrary 97% sequence similarity as cutoff. The OTU richness varied from 691 to 945 per elevational site. Of those sequences, the major phyla were Proteobacteria, Acidobacteria, Bacteroidetes, Chloroflexi, and Planctomycetes (average percentage relative abundance > 5% across all soils), accounting for >85% of the total sequences (Supplementary Figure S2).

**FIGURE 1:**
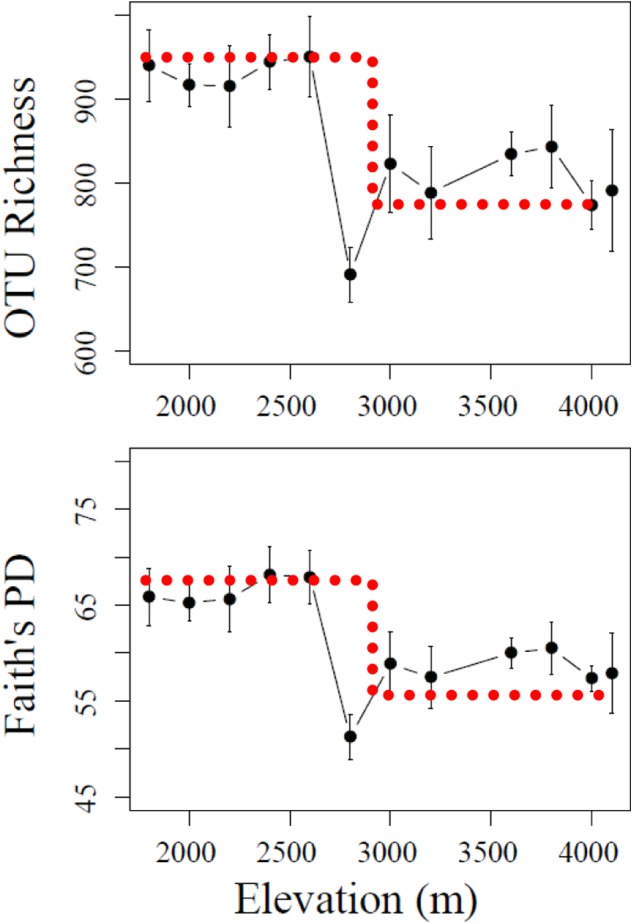
Stair-step patterns of α-diversity indices including OTU richness and Faith’s PD across the elevational gradients. The bar represents standard deviation (SD). The dotted line denotes a “stair-step” shape.

Bacterial abundances, represented by 16S rRNA gene copy number, ranged from 3.2 × 10^9^–1.2 × 10^10^ copies g^-1^ dry soil, but no obvious pattern was observed along the elevational gradients (data not shown). In contrast, α-diversity, estimated by the number of OTUs (richness) and phylogenetic diversity (Faith’s PD), showed a significant drop as elevation increased from 2600 to 2800 m (**Figure 1**). The α-diversity in lower section (1800–2600 m) showed no significant variation (*P* > 0.05), while at higher elevational section, significant differences were mainly found between 2800 m and the other elevations (*P* < 0.001). Therefore, 2600–2800 m was a turning point for elevation-related diversity patterns. Such a pattern is similar to a stair-step with upper (lower elevations) and lower (higher elevations) sections not showing significant changes in α-diversity within each section.

**FIGURE 2:**
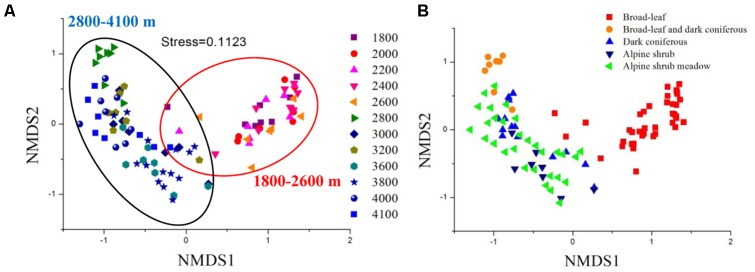
Nonmetric multidimensional scaling (NMDS) analysis of bacterial community compositions from 12 sites across the elevational gradients, ranked by elevation **(A)** and vegetation type **(B)**. The compositional variation is represented by Jaccard distance based on the relative abundances of OTUs.

Bacterial communities significantly changed with elevation, except for several neighboring sites (Supplementary Table S4). The NMDS ordination showed two distinct clusters mainly separated by elevational level (Adonis test, *F* = 9.183, *P* = 0.001; ANOSIM test, *R* = 0.774, *P* = 0.001; MRPP, δ = 0.624, *P* = 0.001) (**Figure 2A**). In addition, the communities were also separated between broad-leaf forests and the other four vegetation types (**Figure 2B**).

### Environmental Determinants of Bacterial Diversity

All available environmental attributes except ${\mathrm{NH}}_{4}^{+}$-N, EB, and DC were significantly correlated with α-diversity (**Table 1**). The richness and Faith’s PD were negatively related to elevation and MAP, while positively correlated with soil pH, ${\mathrm{NO}}_{3}^{-}$-N, TN, TC, T10, soil electric conductivity, PSR, and DB. Among these environmental attributes, pH was the best predictor of bacterial richness (rho = 0.88, *P* < 0.01). Instead, TC and TN were the most significant attributes affecting bacterial abundances (rho = 0.60 and 0.58 for TC and TN, respectively). Partial Mantel tests and CCA results together indicated that soil pH and electric conductivity, PSR, and DB were the most important attributes shaping bacterial community structure (*P* < 0.001) (**Table 1** and Supplementary Figure S3). The importance of these environmental attributes was further verified by Mantel tests (**Table 1**). The results suggested that multiple factors including soil, vegetation, and climate could impose direct or indirect effects on microbial communities across the whole elevational gradients.

**Table 1:** Spearman rank correlation analysis and Mantel test showing the relationships of bacterial α-diversity, the 16S rRNA gene copy number, and bacterial community structure with environmental attributes.

Environmental attribute	Richness^a^	Faith’s PD	16S rRNA gene copy number	Bacterial community structure^b^
Elevation	-0.61**	-0.59**	-0.24*	-0.22
pH	0.88**	0.90**	-0.02	0.84**
${\mathrm{NH}}_{4}^{+}$-N	0.06	0.00	0.44**	-0.02
${\mathrm{NO}}_{3}^{-}$-N	0.64**	0.64**	0.43**	0.2**
TN^C^	0.36**	0.32**	0.58**	0.16**
TC	0.36**	0.32**	0.60**	0.17**
T10	0.60**	0.58**	0.25**	0.19**
Cond.	0.57**	0.56**	0.52**	0.35**
MAP	-0.60**	-0.58**	-0.26**	-0.30
MAT	0.60**	0.58**	0.26**	-0.36
PSR	0.55**	0.53**	0.38**	0.24**
DB	0.61**	0.59**	0.38**	0.30**
EB	-0.09	-0.06	-0.37**	-0.25
DC	-0.14	-0.18	0.19	0.11**

*^a^Spearman rank correlation analysis was conducted for Richness, Faith’s PD, and 16S rRNA gene copy number [number (g dry wt soil)^-1^]. ^b^Partial Mantel tests were used. ^c^Abbreviations: ${\mathrm{NH}}_{4}^{+}$-N [mg (kg dry wt soil)^-1^], ammonium nitrogen [mg (kg dry wt soil)^-1^]; ${\mathrm{NO}}_{3}^{-}$-N, nitrate nitrogen [mg (kg dry wt soil)^-1^]. TN, total nitrogen (%); TC, total carbon (%); T10, soil temperature at 10 cm depth (°C); Cond., soil electric conductivity (μS cm^-1^); MAP, mean annual precipitation (mm); MAT, mean annual air temperature (°C); PSR, plant species richness; DB, EB, and DC represent the percentage of total DBH for DB trees, EB trees, and DC trees, respectively. ^∗^P < 0.05; ^∗∗^P < 0.01.*

**FIGURE 3:**
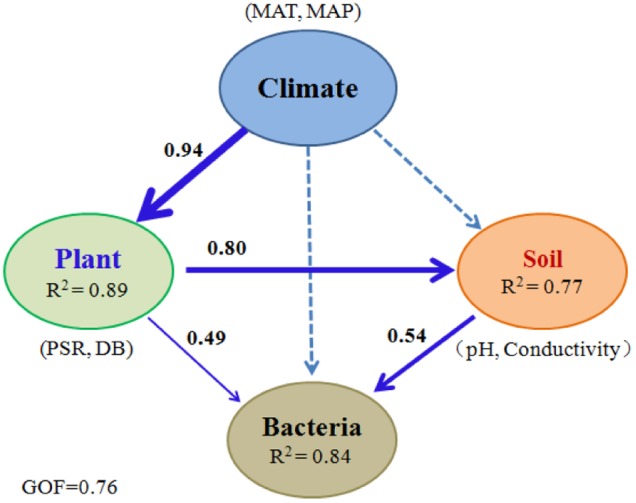
Direct and indirect effects of climate, vegetation, and soil attributes on bacterial communities. PLS-PM was performed for bacterial communities in 99 soil samples collected along the elevational gradients. The width of arrows is proportional to the strength of path coefficients. Continuous and dashed arrows indicate significant and nonsignificant relationships, respectively. *R*^2^ denotes the proportion of variance explained. MAT, mean annual air temperature; MAP, mean annual precipitation; PSR, plant species richness; DB, the percentage of total DBH for DB trees.

To minimize the confounding interactions among causal factors, PLS-PM was implemented to further reveal the possible pathways through which environmental attributes structure microbial communities along the spatial gradients. The model indicated the best fit to the data with GOF = 0.76. The results showed that 84% of the variation in the community structure was accounted for along the sampling sites (**Figure 3**). Climate (MAT and MAP) directly influenced vegetation (mainly PSR and DB) significantly with a higher explained variation (89%). Vegetation could shape microbial community structure directly or indirectly by its effect on soil attributes (mainly soil pH and electric conductivity). However, the direct effect of underground soil attributes on the communities (path coefficient = 0.54) was greater than that of aboveground vegetation (path coefficient = 0.49). All these results suggested that climate indirectly impacted bacterial communities via its effect on both vegetation and soil properties.

### Bacterial Community Assembly Processes

Negative relationship between bacterial community similarity and geographic distance, known as distance-decay of similarity (DDS), was confirmed along the elevational gradients (Supplementary Figure S4A). The slope of DDS at higher elevational section was steeper than that of the lower one (Supplementary Figures S4B,C). Because DDS reflects the combination effect of deterministic and stochastic processes, the difference again implies that mechanisms shaping community assembly between the two elevational sections might be different. Thus, the null model approach was used to discern the relative roles of deterministic versus stochastic process in community assembly. For both elevational sections, the observed Jaccard’s similarities were significantly deviated from the expected ones (**Table 2**), indicating that deterministic processes could play prominent roles in shaping bacterial β-diversity across the sampling sites.

**Table 2:** Jaccard distance-based significance tests of centroid differences between the observed communities and the null model simulations for each elevation using PERMDISP.

Elevation	Centroid of actual communities	Centroid of null model	*F*	*P*
1800	0.405	0.462	49.280	0.001
2000	0.405	0.467	122.816	0.001
2200	0.408	0.468	66.828	0.001
2400	0.405	0.462	65.773	0.001
2600	0.405	0.456	44.478	0.001
2800	0.402	0.515	701.803	0.001
3000	0.405	0.482	129.470	0.001
3200	0.403	0.490	102.176	0.001
3600	0.404	0.485	151.483	0.001
3800	0.414	0.497	304.896	0.001
4000	0.402	0.498	578.417	0.001
4100	0.401	0.495	189.380	0.001

**FIGURE 4:**
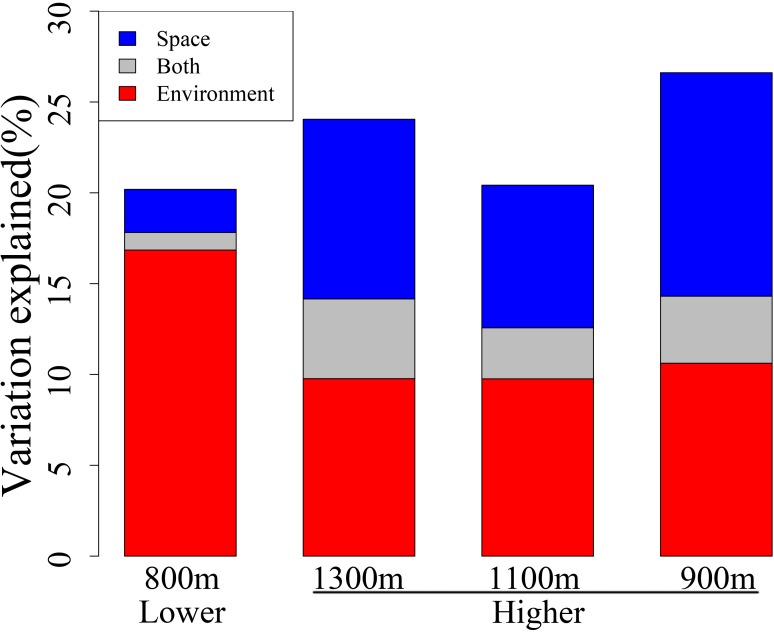
β-Deviation explained by environmental and spatial attributes at different elevational sections, with forward selection model applied in a distance-based redundancy analysis (dbRDA). The scale of lower elevational section was 800 m (1800–2600 m) and scales of higher elevational section were defined as 1300 m (2800–4100 m), 1100 m (3000–4100 m), or 900 m (3200–4100 m), respectively.

To further infer the elevation-related assembly mechanism of bacterial community, we examined the extent to which community compositions varied across environmental and spatial gradients between the two elevational sections using dbRDA. The relative influence of environmental and spatial attributes differed significantly between the lower and higher sections (**Figure 4**). At lower elevations, a much larger fraction of the β-deviation was explained by environmental attributes (16.85%) than by spatial attributes (2.37%) (bootstrap test, *P* < 0.001). However, spatial attributes explained an increased proportion (9.89%) of β-deviation at higher elevations, insignificant to the 9.77% for environmental attributes (bootstrap test, *P* = 0.12). Spatially structured environmental attributes explained higher fraction of β-deviation at higher elevations than the lower ones (4.39% versus 0.97%, bootstrap test, *P* < 0.001). The results suggested that the relative importance of spatial attributes is elevation-related and might play an increased role at higher elevations.

## Discussion

Microbial communities play critical roles in nutrient cycling and ecosystem services. Unraveling the drivers and mechanisms of community succession and community assembly in mountain ecosystems are critical for the projections of soil ecological services in response to climate change. In this study, a unique stair-step pattern in bacterial diversity was revealed along the elevational gradients in Gongga Mountain. Bacterial community structure was largely shaped by soil pH, electric conductivity, PSR, and DB. Moreover, an elevation-related role of spatial attributes in the community assembly was revealed.

### Stair-Step Pattern of Soil Bacterial Diversity Along the Elevational Gradients

The unique stair-step pattern observed in this region is inconsistent with patterns reported in other studies about soil microorganisms, including unimodal (Singh et al., 2012a), monotonical (Shen et al., 2015), or non-significant patterns (Fierer et al., 2011; Shen et al., 2013). This is also not in line with the classical unimodal patterns commonly found in either plants or animals (Guo et al., 2013; Sundqvist et al., 2013). However, since the comprehensive meta-data including climate, vegetation, and soil properties were involved in the analysis, as well as the broader and more finer-grained spatial extent during sampling, our findings could make fundamental contributions to the mechanistic understanding in microbial diversity patterns in mountain ecosystems.

Previous studies suggest that soil pH is a major factor determining microbial diversity and community compositions (Shen et al., 2013; Tripathi et al., 2015; Xia et al., 2016). Lower pH value usually results in lower microbial diversity (Fierer and Jackson, 2006; Lauber et al., 2009; Hollister et al., 2010; Xia et al., 2016). In this study, the elevation pattern of soil pH is generally coincidence with that of bacterial diversity. Moreover, pH 6.0 was observed to be the threshold at which soil pH differed between lower (1800–2600 m) and higher (2800–4100 m) sections. The results showed minor difference with a recent report that pH 5 probably could be as the threshold below which soil bacterial diversity might decline and bacterial community structure might change significantly (Xia et al., 2016). Nonetheless, pH 6.0 is also observed to be the turning point that significant reduction in bacterial diversity occurs when it is lower than 6.0 in grassland ecosystems (Yao et al., 2014). Hence, pH 6.0 possibly acts as the critical point affecting bacterial community compositions in mountain and grassland ecosystems. It is believed that any deviation from neutral pH is supposed to impose stresses on single-cell organism, thus altering overall microbial compositions. Indeed, the phyla Proteobacteria, Bacteroidetes, and Nitrospirae showed significant and positive relationships with soil pH, while Acidobacteria showed negative correlation, which suggests their different tolerance to pH shift.

Apart from soil pH variations, vegetation types significantly shifted from 2600 (broad-leaved forests) to 2800 m (mixed coniferous and broadleaved forests) in this study (**Figure 2B**). In addition, PSR and DB were both significantly correlated with bacterial diversity and community structure. Therefore, bacterial community compositions could be partially related to plant characteristics along the elevational gradients. This is consistent with recently reports that microbial communities differ greatly between vegetation types (Zhang et al., 2015; Veen et al., 2017). Plant traits can modify soil microbial habitat by altering substrate availability (Orwin et al., 2010; Pei et al., 2016), pH (Thoms et al., 2010), and soil moisture (Brussaard et al., 2007). In particular, the amount and quality of plant resource inputs including both aboveground (litter) and belowground (root exudation) materials, e.g., carbon and nitrogen resources, can influence microbial community compositions (Orwin et al., 2010; Chaparro et al., 2014; Kaiser et al., 2015). Indeed, our PLS pathway analysis indicated that plant characteristics had significant direct and indirect effects on microbes. The indirect effects could be accomplished via altering soil physiochemical condition. However, it was worth noting that there were no apparent segregations among the other three vegetation types (3000–4100 m). This could be attributed to the overwhelming effect of pH on microbial communities, since soil pHs in the other three vegetation types all fell below 6.0, which might mask the effects of vegetation types.

### Roles of Environmental Filtering and Spatial Attributes in the Assembly of Bacterial Communities

Understanding mechanisms of microbial community assembly is a central issue in community ecology (Chase, 2003; Zhou et al., 2014). The analysis of β-diversity has been widely applied for exploring community assembly along ecological gradients (Adams et al., 2013). In this study, distinct differences in β-diversity and DDS pattern between the two elevational sections imply that mechanism dominating the community assembly in the two sections might be different. However, the results obtained by null model analysis supported for a prominent role of deterministic environmental filtering in shaping the succession of soil bacterial communities at both lower and higher elevations. Since environmental attributes correlated not only with bacterial α- and β-diversity, but also with specific bacterial phyla (Supplementary Table S5), these abiotic variables are expected to exert significant filtering effects on bacterial communities, in line with the niche theory.

Interestingly, further in-depth analysis showed that, although strong environmental filtering predominates the whole spatial region, the pure spatial attributes dramatically increased to much higher level (13.87%) at higher elevations, which suggests an elevation-related role of spatial attributes. However, the lower and higher elevational sections of this study region expanded approximately equal horizontal distance (7 km), which excluded the horizontal scale effects. Moreover, it is not likely that the observation was due to different elevational scale between the lower (800 m) and higher (1300 m) section, because spatial attributes still explained 8.59 and 9.71% variations when higher section occupied 1100 (3000–4100 m) and 900 m (3200–4100 m), respectively, which were both much higher than that of lower section (2.44%, **Figure 4**). Thus, the scale effect should not be the cause.

Alternatively, environmental heterogeneity might account for this phenomenon. This is supported by the fact that the distance-to-centroid values for environmental attributes in higher elevations were significantly higher than those of lower elevations (Supplementary Figure S5), which suggests a higher heterogeneous habitat in the higher section. Therefore, higher environmental heterogeneity in the higher elevations will result in a more importance of spatial attributes in bacterial community assembly. However, at higher elevations, the effect of spatial attributes did not exceed than that of environmental attributes, possibly because of the pivotal effect of environmental filtering on microbial communities in the whole mountain region. Further, different habitat type might be another reason. It is reported that the relative importance of environmental filtering and geographic distance varies across habitat types (Wang X.-B. et al., 2017). For example, geographic distance was solely responsible for community similarity in the desert, while in the alpine grassland it was influenced only by the environmental factors. In this study, the lower elevational section is predominantly occupied by broadleaved forests, while the higher elevational section is covered by several habitat types, such as conifer forests and alpine shrubs. The difference in habitat type between the two elevational sections might influence bacterial community assembly. The underlying mechanisms remain to be revealed.

## Conclusion

Unraveling microbial diversity patterns in mountain ecosystem, as well as environmental drivers and species assembly mechanism, are critical for the projections of soil ecological services in response to climate change. In this study, we observed a stair-step pattern of soil bacterial diversity along the elevational gradients of Gongga Mountain. The unique pattern was primarily shaped by soil pH and vegetation types. Our PLS-PM results imply that climate change, e.g., MAP and MAT variation, will probably not directly affect bacteria itself in mountain ecosystems, but through indirect interacting effects of plant and soil attributes. Interestingly, although environmental filtering dominated at all elevation, spatial attributes showed an increased role at higher elevations. These results highlight the importance of environmental filtering, as well as elevation-related spatial attributes in structuring soil bacterial communities in mountain ecosystems. However, it is noteworthy that our findings are based on 16S rRNA genes, and the functioning responses to environment changes mediated by microbial communities urges further studies.

## Author Contributions

JL and XL designed the experiments. JL, BT, and SZ collected the soil samples. ZS contributed to the plant survey. JL, CL, YK, and YW performed the experiments. JL wrote the paper. XL, JL, and ZS revised the paper.

## Conflict of Interest Statement

The authors declare that the research was conducted in the absence of any commercial or financial relationships that could be construed as a potential conflict of interest.

## Footnotes

1 http://www.ebi.ac.uk/ena/data/view/PRJEB15866
