# Supplementary material for: Stair-Step Pattern of Soil Bacterial Diversity Mainly Driven by pH and Vegetation Types Along the Elevational Gradients of Gongga Mountain, China
Source: Front Microbiol. 2018 Mar 27;9:569. doi: 10.3389/fmicb.2018.00569 (PMC5880914; doi:10.3389/fmicb.2018.00569)
Supplement: Supplementary file 1 [file Data_Sheet_1.docx]

| **Supporting information**  Additional Supporting Information may be found in the  online version of this article at the publisher’s web-site:  Additional Supporting Information may be found in the  online version of this article at the publisher’s web-site:  Additional Supporting Information may be found in the  online version of this article at the publisher’s web-site:  Additional Supporting Information may be found in the online version of this article at the publisher's web-site:  **Table S1.** The plant attributes for each elevational site. | | | | |
| --- | --- | --- | --- | --- |
| Elevation (m) | PSR^a^ | DB (%) | EB (%) | DC (%) |
| 1800 | 110.00 | 79.40 | 20.20 | 0.37 |
| 2000 | 92.33 | 49.20 | 50.80 | 0.00 |
| 2200 | 90.33 | 80.83 | 19.17 | 0.00 |
| 2400 | 82.00 | 57.87 | 40.03 | 2.10 |
| 2600 | 74.00 | 57.33 | 6.83 | 35.87 |
| 2800 | 64.33 | 17.20 | 2.77 | 80.03 |
| 3000 | 55.00 | 8.87 | 1.77 | 89.37 |
| 3200 | 42.67 | 4.87 | 11.63 | 83.50 |
| 3600 | 25.33 | 1.37 | 67.73 | 30.93 |
| 3800 | 36.00 | 0.00 | 100.00 | 0.00 |
| 4000 | 41.33 | 0.00 | 100.00 | 0.00 |
| 4100 | 40.67 | 0.00 | 100.00 | 0.00 |
| ^a^ PSR, plant species richness; DB, EB and DC represent the percentage of total DBH for deciduous broad trees, evergreen broad trees, and dark coniferous trees, respectively. | | | | |

| **Table S2.** Soil properties along the elevational site. The values are shown as mean ± standard deviation. | | | | | | | |
| --- | --- | --- | --- | --- | --- | --- | --- |
| Elevation | pH | NH_4_^+^-N | NO_3_^-^-N | TN | TC | T10 | Cond. |
| 1800 | 6.20±0.60 | 12.58±7.77 | 18.73±5.15 | 0.99±0.50 | 11.72±6.52 | 14.44±1.67 | 115.35±55.40 |
| 2000 | 6.41±0.49 | 16.9±10.02 | 6.51±7.26 | 1.11±0.50 | 15.37±8.70 | 13.23±0.31 | 145.98±85.52 |
| 2200 | 6.14±0.62 | 18.39±10.23 | 15.54±8.71 | 1.48±0.40 | 21.20±7.78 | 11.30±0.13 | 152.78±52.08 |
| 2400 | 6.63±0.53 | 16.05±12.40 | 4.18±5.01 | 0.99±0.59 | 14.51±7.04 | 10.35±0.27 | 166.20±68.76 |
| 2600 | 6.25±0.34 | 20.70±13.95 | 12.31±11.09 | 1.23±0.71 | 17.61±11.44 | 10.43±0.48 | 157.31±91.89 |
| 2800 | 4.16±0.31 | 24.29±10.27 | 0.28±0.33 | 0.99±0.42 | 13.94±7.84 | 8.78±0.34 | 62.35±26.37 |
| 3000 | 4.80±0.45 | 36.62±18.06 | 0.11±0.13 | 0.70±0.21 | 9.10±3.11 | 6.87±0.48 | 58.40±33.19 |
| 3200 | 4.77±0.44 | 33.22±5.74 | 1.19±3.03 | 0.64±0.20 | 8.84±3.66 | 6.39±0.18 | 45.71±17.79 |
| 3600 | 5.06±0.47 | 11.59±19.91 | 0.06±0.10 | 0.37±0.58 | 6.17±11.39 | 4.78±0.71 | 39.77±43.30 |
| 3800 | 5.40±0.30 | 9.06±9.60 | 0.08±0.15 | 0.37±0.28 | 4.28±3.69 | 5.71±0.61 | 34.17±20.76 |
| 4000 | 4.81±0.27 | 6.20±3.90 | 0.21±0.43 | 0.44±0.20 | 5.13±2.27 | 4.59±0.60 | 35.30±10.43 |
| 4100 | 4.95±0.35 | 5.32±1.28 | 0.49±0.41 | 0.56±0.13 | 6.29±1.67 | 3.79±0.49 | 45.51±15.76 |

| **Table S3.** Spearman rank correlation analysis showing the relationships between each environmental attribute. | | | | | | | | | | | | | |
| --- | --- | --- | --- | --- | --- | --- | --- | --- | --- | --- | --- | --- | --- |
|  | Elevation | pH | NH_4_^+^-N | NO_3_^-^-N | TN | TC | T10 | Cond. | MAP | MAT | PSR | DB | EB |
| pH | -0.58** |  |  |  |  |  |  |  |  |  |  |  |  |
| NH_4_^+^-N | -0.36** | -0.13 |  |  |  |  |  |  |  |  |  |  |  |
| NO_3_^-^-N | -0.66** | 0.62** | 0.16 |  |  |  |  |  |  |  |  |  |  |
| TN^a^ | -0.58** | 0.24* | 0.74** | 0.57** |  |  |  |  |  |  |  |  |  |
| TC | -0.60** | 0.23* | 0.76** | 0.55** | 0.98** |  |  |  |  |  |  |  |  |
| T10 | -0.96** | 0.58** | 0.37** | 0.64** | 0.61** | 0.62** |  |  |  |  |  |  |  |
| Cond. | -0.67** | 0.50** | 0.57** | 0.64** | 0.87** | 0.88** | 0.68** |  |  |  |  |  |  |
| MAP | 0.99** | -0.57** | -0.37** | -0.66** | -0.59** | -0.60** | -0.96** | -0.66** |  |  |  |  |  |
| MAT | -0.99** | 0.57** | 0.37** | 0.66** | 0.59** | 0.61** | 0.96** | 0.66** | -1.00** |  |  |  |  |
| PSR | -0.90** | 0.52** | 0.34** | 0.72** | 0.63** | 0.64** | 0.91** | 0.70** | -0.90** | 0.90** |  |  |  |
| DB | -0.94** | 0.55** | 0.37** | 0.69** | 0.62** | 0.65** | 0.89** | 0.71** | -0.93** | 0.93** | 0.88** |  |  |
| EB | -0.67** | 0.49** | 0.07 | 0.37** | 0.22* | 0.28** | 0.54** | 0.36** | -0.66** | 0.66** | 0.41** | 0.59** |  |
| DC | -0.19 | -0.30** | 0.51** | -0.14 | 0.08 | 0.14 | 0.09 | 0.05 | -0.19 | 0.19 | 0.05 | 0.23* | 0.10 |
| ^a^ Abbreviations: TC, total carbon; T10, soil temperature at 10 cm depth; Cond., soil electric conductivity; MAP, mean annual precipitation; MAT, mean annual air temperature; PSR, plant species richness; DB, EB and DC represent the percentage of total DBH for deciduous broad trees, evergreen broad trees, and dark coniferous trees, respectively. * *P*< 0.05; ** *P*< 0.01. The units are listed in Table 1. | | | | | | | | | | | | | |

| **Table S4.** Nonparametric multivariate permutation test (Adonis) showing the differences of bacterial communities between each elevational site. | | | | | | | | | | | |
| --- | --- | --- | --- | --- | --- | --- | --- | --- | --- | --- | --- |
| Elevation | 2000 | 2200 | 2400 | 2600 | 2800 | 3000 | 3200 | 3600 | 3800 | 4000 | 4100 |
| 1800 | 0.99^a^ | 1.08 | 1.22* | 1.1 | 3.51** | 2.15** | 2.4** | 2.56** | 2.59** | 3.02** | 2.85** |
| 2000 |  | 1.21* | 1.21* | 1.18* | 3.91** | 2.43** | 2.76** | 2.76** | 2.96** | 3.37** | 3.11** |
| 2200 |  |  |  | 1.01 | 3.52** | 2.06** | 2.44** | 2.55** | 2.57** | 2.97** | 2.83** |
| 2400 |  |  |  | 1.01 | 3.89** | 2.46** | 2.79** | 2.89** | 3.02** | 3.36** | 3.28** |
| 2600 |  |  |  |  | 3.38** | 2.08** | 2.36** | 2.36** | 2.36** | 2.9** | 2.69** |
| 2800 |  |  |  |  |  | 1.66** | 1.46** | 2.57** | 2.75** | 1.51** | 1.72** |
| 3000 |  |  |  |  |  |  | 1.02 | 1.38** | 1.17* | 1.32** | 1.21* |
| 3200 |  |  |  |  |  |  |  | 1.51** | 1.43** | 1.14* | 1.11 |
| 3600 |  |  |  |  |  |  |  |  | 1.23** | 1.79** | 1.7** |
| 3800 |  |  |  |  |  |  |  |  |  | 1.77** | 1.55** |
| 4000 |  |  |  |  |  |  |  |  |  |  | 1.03 |
| ^a^ Adonis F value; * *P*< 0.05; ** *P*< 0.01. | | | | | | | | | | | |

| **Table S5.** Spearman rank correlation analysis showing the relationships between environmental attributes and main bacterial phylum. | | | | | | | | | | | | | |
| --- | --- | --- | --- | --- | --- | --- | --- | --- | --- | --- | --- | --- | --- |
| Phylum | Elevation | pH | NO_3_^-^-N | TN^a^ | TC | T10 | Cond. | MAP | MAT | PSR | DB | EB | DC |
| Proteobacteria | -0.45** | 0.50** | 0.32** | 0.25* | 0.27** | 0.45** | 0.37** | -0.45** | 0.45** | 0.27** | 0.41** | 0.39** |  |
| Acidobacteria | 0.60** | -0.87** | -0.60** | -0.30** | -0.29** | -0.57** | -0.50** | 0.58** | -0.58** | -0.48** | -0.53** | -0.50** |  |
| Bacteroidetes | -0.49** | 0.79** | 0.50** |  |  | 0.45** | 0.38** | -0.46** | 0.46** | 0.38** | 0.38** | 0.47** | -0.27** |
| Chloroflexi | 0.48** |  |  |  | -0.23* | -0.45** |  | 0.49** | -0.49** | -0.36** | -0.36** | -0.35** |  |
| Planctomycetes | -0.23* | 0.33** | 0.36** | 0.29** | 0.29** | 0.26* | 0.36** | -0.23* | 0.23* | 0.36** | 0.33** |  |  |
| Actinobacteria | 0.39** | -0.23* | -0.33** | -0.33** | -0.36** | -0.42** | -0.42** | 0.38** | -0.38** | -0.56** | -0.47** |  |  |
| Thaumarchaeota |  |  |  |  |  |  |  |  |  | 0.32** |  |  |  |
| Nitrospirae | -0.49** | 0.76** | 0.59** | 0.26** | 0.23* | 0.51** | 0.42** | -0.48** | 0.48** | 0.51** | 0.54** |  |  |
| Gemmatimonadetes |  | 0.31** |  | -0.23* | -0.24* |  |  |  |  |  |  |  |  |
| ^a^ Abbreviations: TN, total nitrogen; TC, total carbon; T10, soil temperature at 10 cm depth; Cond., soil electric conductivity; MAP, mean annual precipitation; MAT, mean annual air temperature; PSR, plant species richness; DB, EB and DC represent the percentage of total DBH for deciduous broad trees, evergreen broad trees, and dark coniferous trees, respectively. * *P*< 0.05; ** *P*< 0.01. The units are listed in Table 1. Only significant results were shown. | | | | | | | | | | | | | |

**Figure S1.** Nonmetric multidimensional scaling (NMDS) analysis of bacterial community compositions from 12 sites across the elevational gradients. The compositional variation is represented by Jaccard (A) and Bray-Curtis (B) distance based on the relative abundances of OTUs.

B


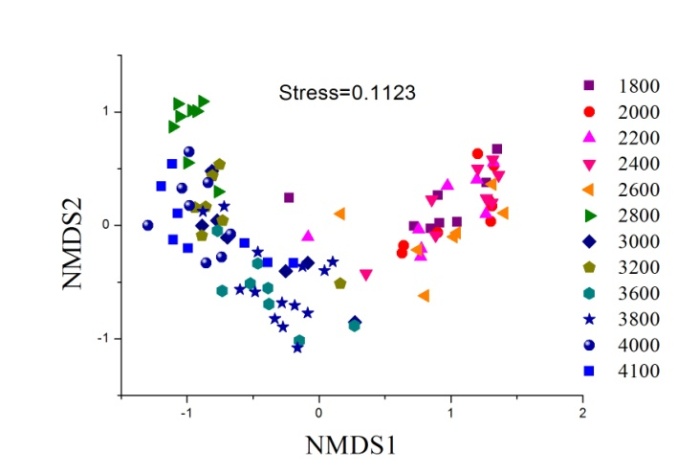

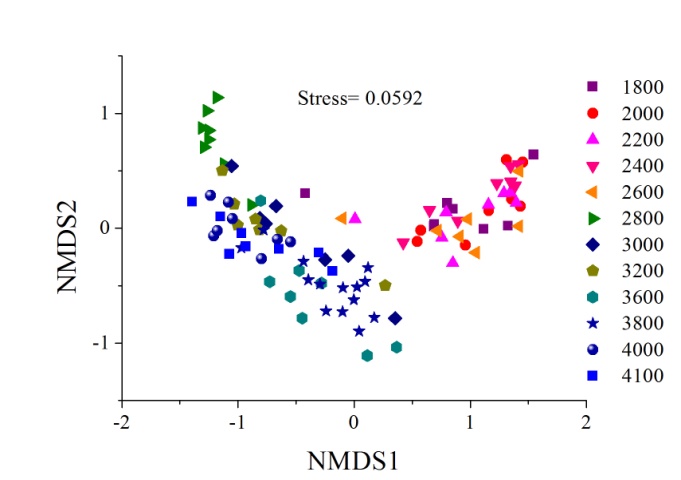


A

**Figure S2.** Percentage relative abundances of main bacterial phyla (> 0.5%) in soils from 12 elevational sites.

**
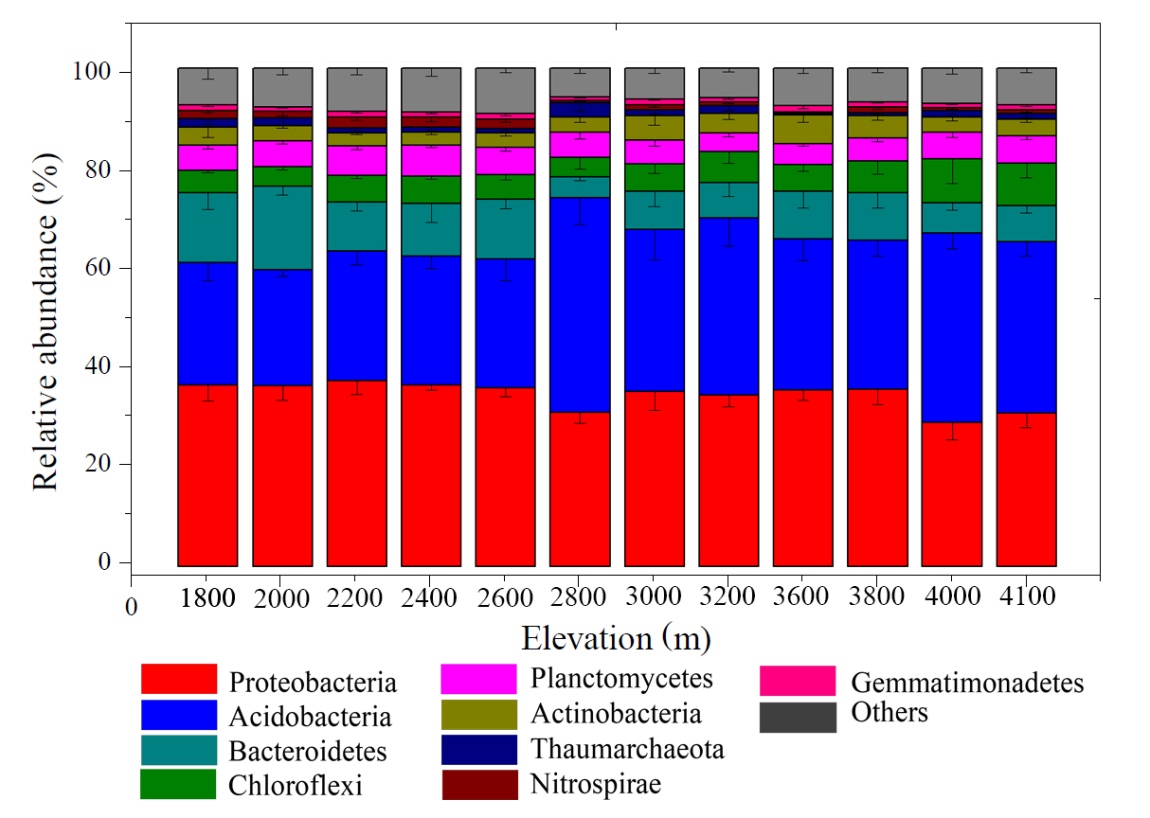
**

**Figure S3.** Canonical correspondence analysis (CCA) showing the relationships of environmental attributes with bacterial community structure. T10, soil temperature at 10 cm depth; MAT, mean annual air temperature; PSR, plant species richness; DB and DC represent the percentages of total DBH for deciduous broad trees, and dark coniferous trees, respectively.

**
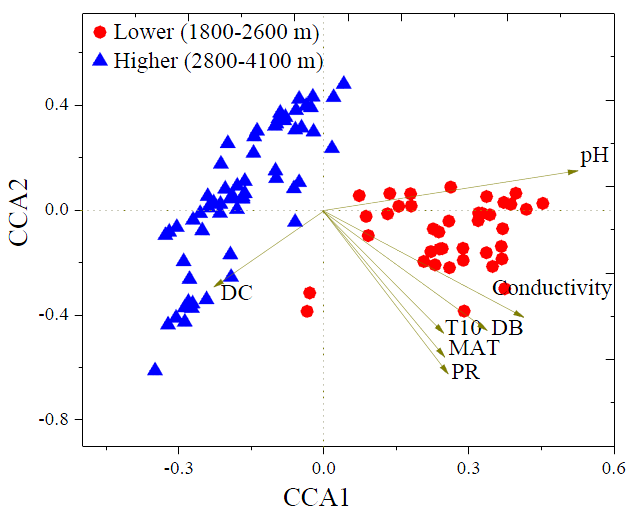
**

**Figure S4.** Relationships between the geographic distances (Km) and bacterial community distances estimated by the 1-Bray-Curits similarity index. All, higher and lower elevations represent elevational scales of 1800-4100 m, 2800-4100 m, and 1800-2600 m, respectively.


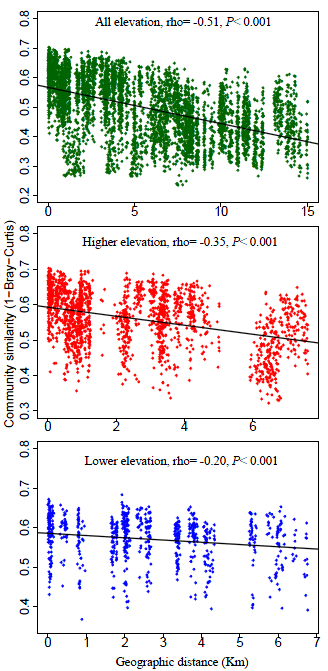


**Figure S5.** Distance-to-centroid values of environmental attributes between the higher (2800-4100 m) and lower (1800- 2600 m) elevations.

**
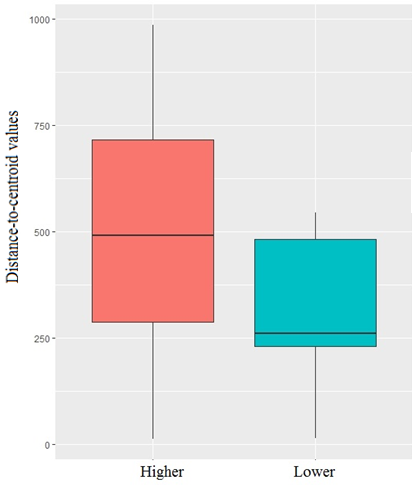
**
